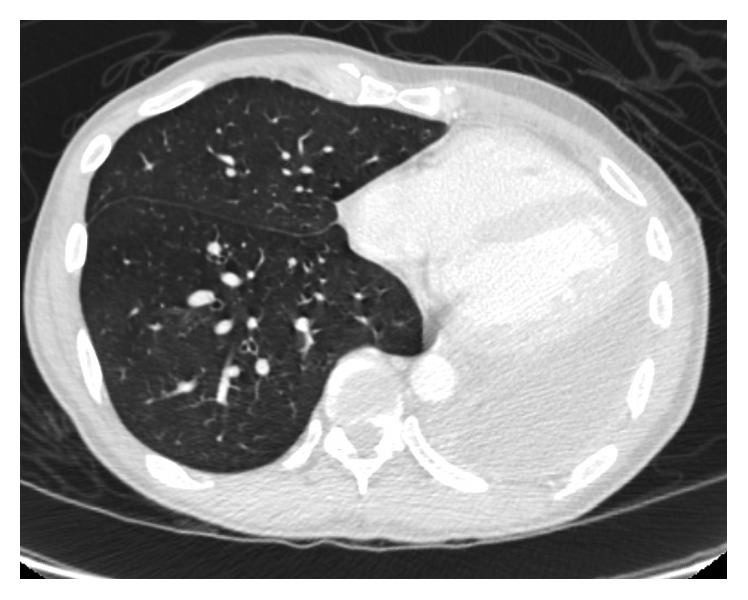# The Infection Returns: A Case of Pulmonary Sporotrichosis Relapse after Chemotherapy

**DOI:** 10.1155/2018/1384029

**Published:** 2018-02-18

**Authors:** Samid M. Farooqui, Houssein Youness

**Affiliations:** ^1^Department of Internal Medicine, University of Oklahoma Health Sciences Center, Oklahoma City, OK, USA; ^2^Section of Pulmonary, Critical Care, and Sleep Medicine, University of Oklahoma Health Sciences Center, Oklahoma City, OK, USA

## Abstract

**Background:**

Pulmonary sporotrichosis is a rare disease caused by a dimorphic fungus, *Sporothrix schenckii*. It is rarely found in association with malignancy. We present a case of pulmonary sporotrichosis recurrence after chemotherapy.

**Case Presentation:**

A 44-year-old man, treated for pulmonary sporotrichosis in the past, presented with dysphagia and was found to have squamous cell carcinoma of the esophagus. After undergoing chemotherapy, extensive cavitary lesions were observed on thoracic computed tomography scan. A bronchoalveolar lavage revealed the presence of *Sporothrix schenckii sensu lato*. Despite treatment with itraconazole, he eventually required a left pneumonectomy for progressive destructive cavitary lesions involving the left lung.

**Conclusion:**

This case highlights the importance of considering past fungal infections, albeit cured, in patients initiating immunosuppressive therapy.

## 1. Background

Pulmonary sporotrichosis is a rare disease caused by *Sporothrix schenckii* [1]. The dimorphic fungus was initially described in 1898 [2]. The primary and multifocal forms of pulmonary infection were described later in 1975 [3]. 88 cases were reported between 1960 and 2013 out of which only one case was related to malignancy. We present a case of pulmonary sporotrichosis that relapsed after starting chemotherapy for esophageal cancer.

## 2. Case Presentation

A 44-year-old African American man presented with a past medical history of pulmonary sporotrichosis treated with itraconazole in 2008 and in 2011 for 5 months at least and with a documented negative sputum culture thereafter. He worked as a transporter for bird seed bags. He has a 20 pack-year history of smoking and drinks three cans of beers three times per week.

**Figure 1:**
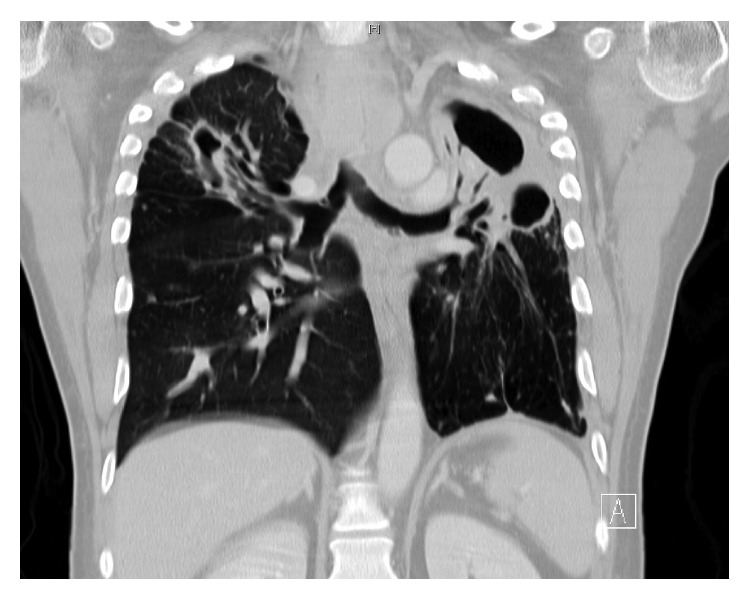


**Figure 2:**
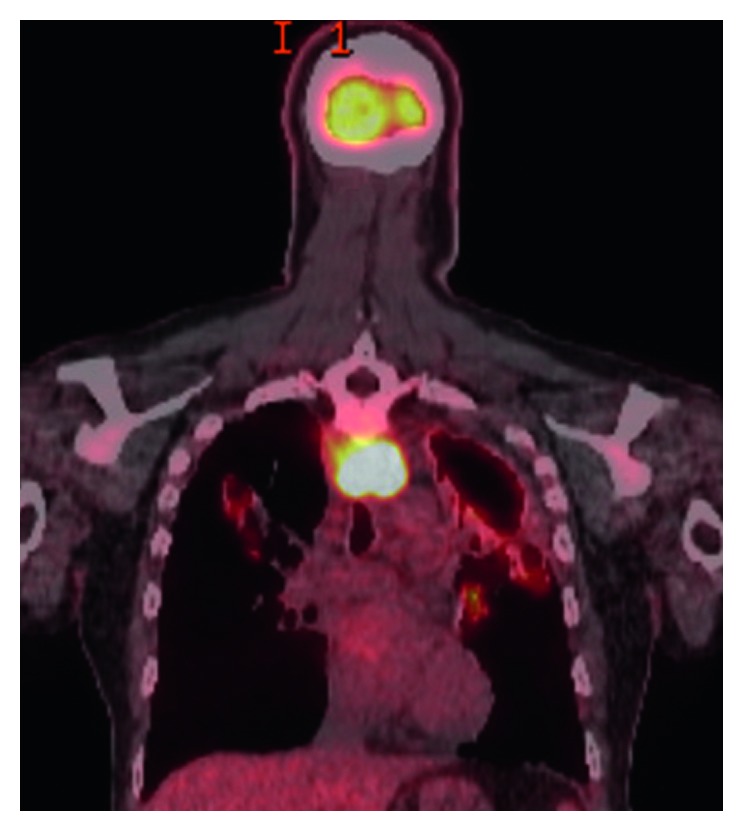


He was asymptomatic from May 2012 till July 2015 when he presented with dysphagia and hemoptysis. CT chest (Figure 1) revealed a retropharyngeal mass concerning abscess, a 35 × 12 × 8 mm right upper lobe (RUL) cavity with larger and multiple cavitations involving the left upper lobe (LUL). There were multiple bilateral small nodules as well, which had low FDG uptake on PET scan (Figure 2).

Incision and drainage of the neck abscess was performed and cultures grew *streptococcus viridians* and *Candida*. Esophageal biopsy was positive for squamous cell carcinoma.

A bronchoscopy with bronchoalveolar lavage (BAL) of the LUL showed neutrophil predominance (88% neutrophils). BAL culture was positive for *alpha hemolytic streptococci*.

Broad-spectrum antimicrobial therapy with vancomycin, metronidazole, levofloxacin, and fluconazole was given for 3 weeks.

After discussion in tumor board, chemotherapy with carboplatin and paclitaxel along with radiation therapy was started in August 2015, both of which were completed in September 2015.

**Figure 3:**
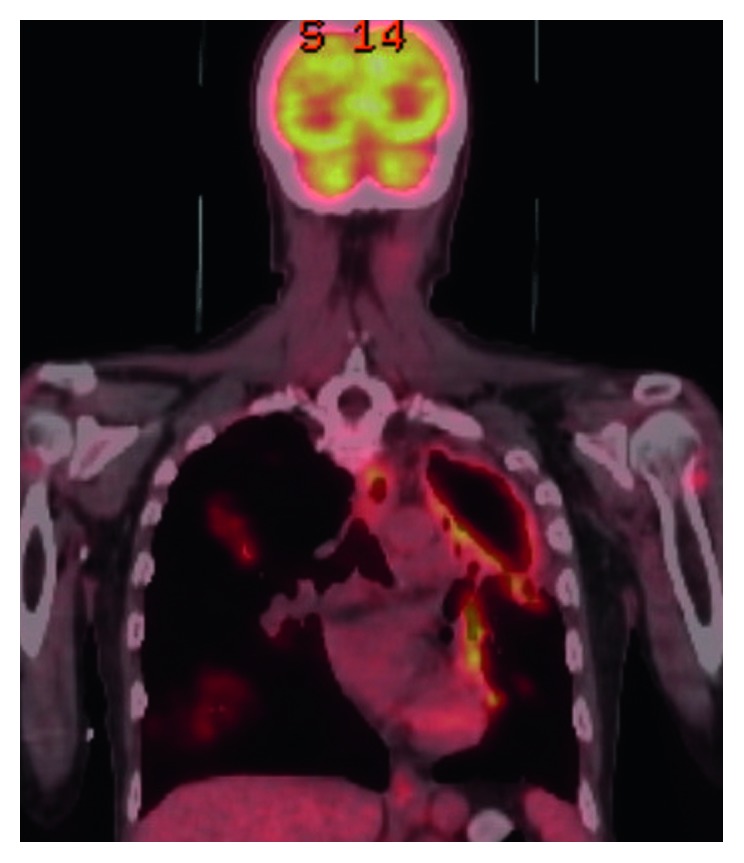


**Figure 4:**
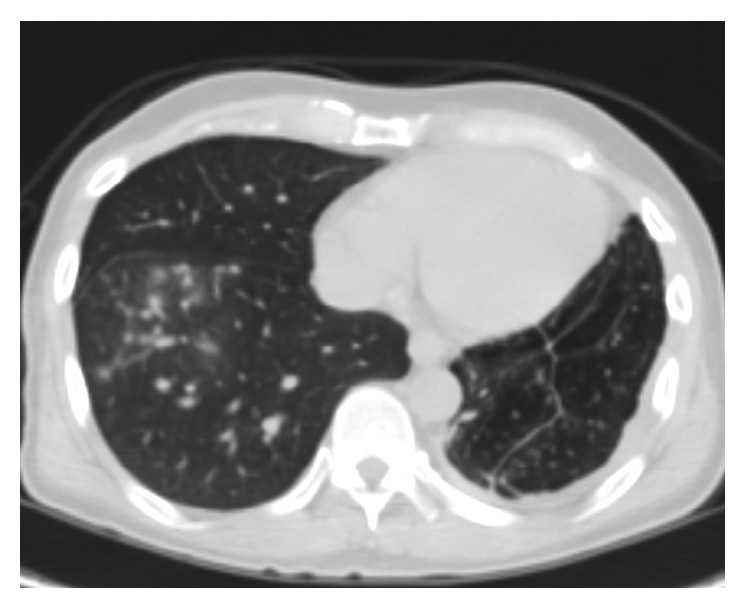


Follow-up imaging with PET scan (Figure 3) and CT scan (Figure 4) in October 2015 revealed marked improvement of the esophageal mass, extensive cavitary pulmonary lesions bilaterally, and an increase in bilateral pulmonary nodules in size, number, and FDG metabolic activities.

A repeat BAL of the right and left lower lobes revealed the presence of *Sporothrix schenckii sensu lato*. Itraconazole at 200 mg twice daily was started. There was difficulty in obtaining frequent itraconazole levels due to noncompliance. Serum itraconazole level measured in May 2016 was adequate though at 1.0 mcg/mL (normal ≥ 0.6 mcg/mL).

**Figure 5:**
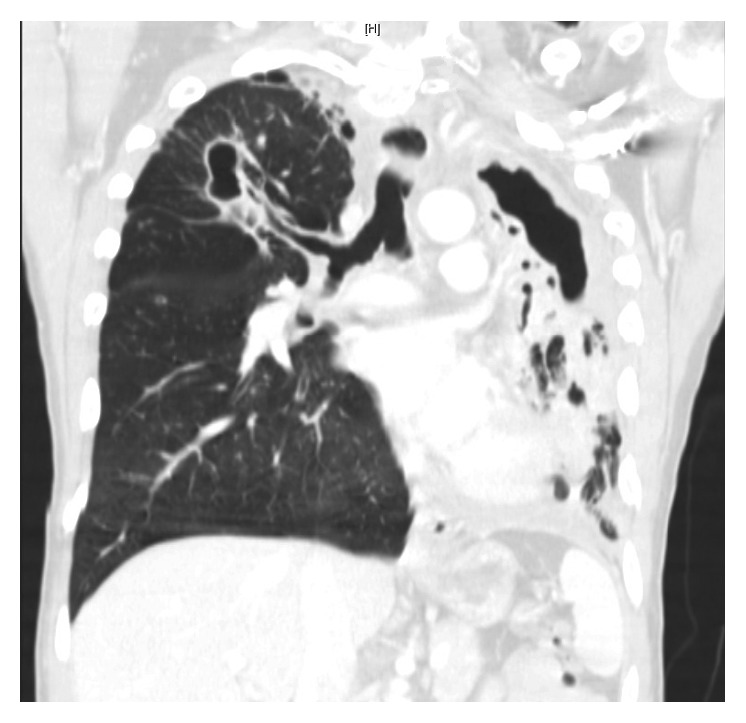


**Figure 6:**
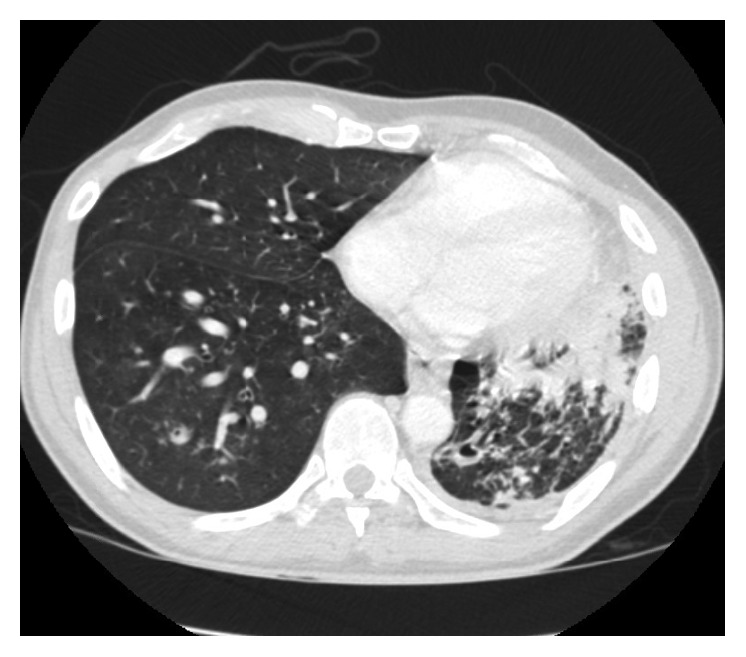


Repeat CT chest (Figures 5 and 6) in August 2016 revealed worsening destructive cavitary changes involving mainly the left lung and the right-sided pulmonary nodules increased in size and some of them started to cavitate.

**Figure 7:**
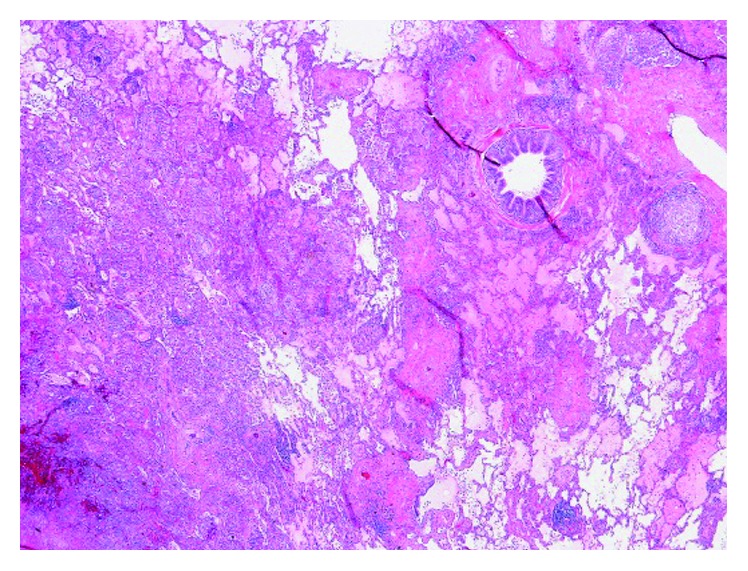
Pulmonary sporotrichosis: ×2 magnified view of the caseating necrosis.

**Figure 8:**
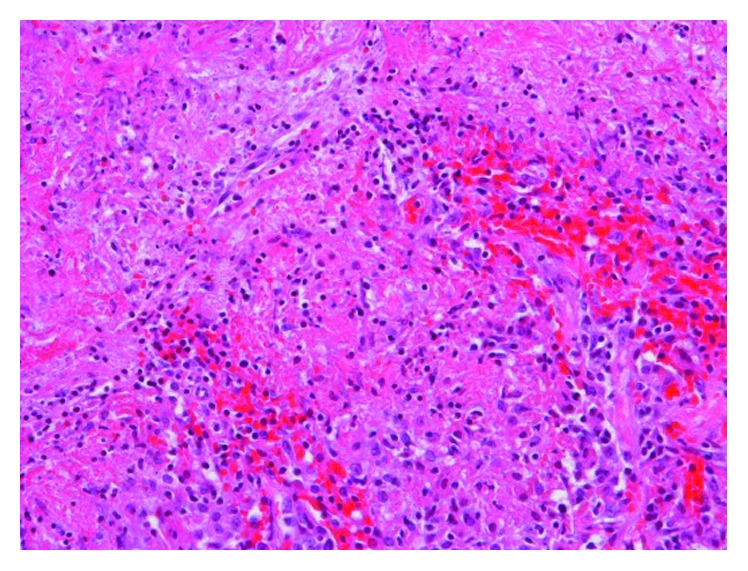
Pulmonary sporotrichosis: ×20 magnified view of the caseating necrosis.

Serum itraconazole level was repeated and found to be subtherapeutic (<0.1 mcg/mL; normal ≥ 0.6 mcg/mL). Since the itraconazole level was subtherapeutic, the itraconazole dose was increased to 200 mg PO three times a day, after consulting with infectious diseases. However, due to the significant extent of the destructive changes involving the left lung and since a ventilation perfusion scan revealed that the left lung perfusion was down to 2% only, a left pneumonectomy was performed in August 2016 and the patient was discharged on itraconazole. The microbiological cultures from the resected specimen grew *Sporothrix schenckii sensu lato* (via yeast API® and via morphology on a lactophenol cotton blue scotch tape prep). Pathology (Figures 7 and 8) showed the presence of numerous granulomas, most with caseating necrosis and was consistent with *Sporothrix* infection.

**Figure 9:**
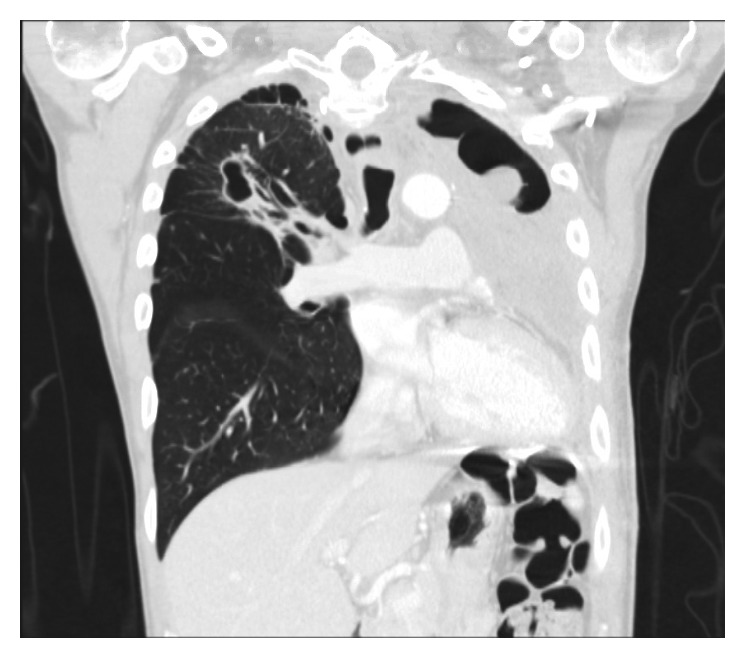


A repeat CT chest (Figures 9 and 10) in September 2016 showed resolution of the RLL cavitary lesions and interval improvement in the RLL nodules.

## 3. Discussion

Sporotrichosis is a rare disease caused by the dimorphic fungus *Sporothrix Schenckii sensu lato* [4], and molecular studies have shown that this species is made up of a complex of 6 different phylogenetic species. These include *Sporothrix schenckii sensu stricto*, *Sporothrix brasiliensis*, *Sporothrix globosa*, *Sporothrix mexicana*, *Sporothrix pallida*, *and Sporothrix luriei*. *Sporothrix schenckii sensu stricto and Sporothrix brasilensis* are the most common causes of infections in humans [5].

It can affect multiple organ systems in the body, with lymphocutaneous sporotrichosis being the most common presentation [6]. Pulmonary infections can occur in isolation as it occurs in primary pulmonary sporotrichosis (PPS) or in the setting of multiorgan involvement as in the setting of multifocal sporotrichosis (MFS) [7].

A total of 88 cases of pulmonary sporotrichosis were reported between 1960 and 2013 [7].

Male gender, middle age, underlying pulmonary disease, immunosuppression, and alcoholism are considered risk factors [6, 7]. Only one case of meningeal MFS has been reported to occur in a patient with Hodgkin's lymphoma [8]. Our case is the first reported case of PPS associated with malignancy with recurrence after chemotherapy for esophageal cancer.

Traumatic inoculation is the mode of introduction of fungus in the cutaneous form [9]. Occupational or recreational contact with plant materials (flowers, roses, and blackberries), animals (horses and deer), and soil particles (brickmason and gardening) has been reported for pulmonary sporotrichosis [7]. In our case, the only potential exposure reported by the patient was that of transporting bird seed bags.

Patients generally present with constitutional signs and symptoms. In PPS, the diagnosis is established by the culture of sputum and bronchoscopic specimen, whereas tissue samples of skin and joints establish the diagnosis in the majority of MFS [10].

Radiologically, pulmonary sporotrichosis can present as cavitary or noncavitary depending on whether the infection is primary pulmonary (PPS) or multifocal sporotrichosis (MFS). Most patients with primary cavitary disease present with unilobular upper lobe involvement as opposed to multilobular reticulonodular infiltrates which is more common in the MFS [7]. Note that MFS with pulmonary involvement occurs more commonly in the setting of immunosuppression [6].

Medical treatment with azole medications is the standard of care; however, in severe or life-threatening cases, lipid amphotericin B is recommended as the initial treatment followed by itraconazole for at least 12 months [11]. Further treatment of pulmonary sporotrichosis should be guided by the radiological patterns of presentation. Patients with cavitary changes have a better outcome when surgical resection is added to medical therapy. However, in noncavitary disease, medical therapy alone provides good outcomes [7]. In our case, since there was extensive loss of lung function on the left side, it was decided to proceed with left pneumonectomy. The decision to increase the itraconazole dose to 200 mg PO three times a day instead of using amphotericin B was based on finding a subtherapeutic level of itraconazole with no associated acute hemodynamic compromise or significant oxygen desaturation.

This is a unique case demonstrating recurrence of sporotrichosis after chemoradiotherapy treatment for esophageal squamous cell carcinoma. It underlines the importance of considering recurrence of latent fungal infections in patients receiving antineoplastic treatment. We believe that, in high-risk patients, restarting antifungal treatment should be considered at the time of initiation of antineoplastic treatment.

## Figures and Tables

**Figure 10:**